# A system suitability testing platform for untargeted, high-resolution mass spectrometry

**DOI:** 10.3389/fmolb.2022.1026184

**Published:** 2022-10-11

**Authors:** Andrei Dmitrenko, Michelle Reid, Nicola Zamboni

**Affiliations:** ^1^ Institute of Molecular Systems Biology, Department of Biology, ETH Zürich, Zürich, Switzerland; ^2^ Life Science Zurich PhD Program on Systems Biology, Zürich, Switzerland

**Keywords:** metabolomics, quality assurance, quality control, mass spectrometry, analytical chemistry

## Abstract

The broad coverage of untargeted metabolomics poses fundamental challenges for the harmonization of measurements along time, even if they originate from the very same instrument. Internal isotopic standards can hardly cover the chemical complexity of study samples. Therefore, they are insufficient for normalizing data *a posteriori* as done for targeted metabolomics. Instead, it is crucial to verify instrument’s performance *a priori*, that is, before samples are injected. Here, we propose a system suitability testing platform for time-of-flight mass spectrometers independent of liquid chromatography. It includes a chemically defined quality control mixture, a fast acquisition method, software for extracting ca. 3,000 numerical features from profile data, and a simple web service for monitoring. We ran a pilot for 21 months and present illustrative results for anomaly detection or learning causal relationships between the spectral features and machine settings. Beyond mere detection of anomalies, our results highlight several future applications such as 1) recommending instrument retuning strategies to achieve desired values of quality indicators, 2) driving preventive maintenance, and 3) using the obtained, detailed spectral features for posterior data harmonization.

## Introduction

Reproducibility and replicability of experiments are essential mainstays of the scientific method (John, 2017). Failure to reproduce measurements, computations, or results of a previous study is perceived as a lack of rigor and undermines the validity of study and its claims. Omics technologies are not immune to these challenges (Tarazona et al., 2020). In fact, issues tend to increase with time and the steadily increasing number of features that every new technology allows to detect. This exacerbates the problems of small sample size (IntHout et al., 2015) and overfitting. Mass spectrometry (MS)-based assays also suffer from the inherent variability of measurements across instruments and over time. In proteomics, lipidomics, metabolomics, etc., reproducibility of quantitative experiments is a well-known issue (Benton et al., 2012; Martin et al., 2015; Beger et al., 2019). The common workaround to enable quantitation in MS-based assays is to add a known amount of isotopically labeled internal standards (IS), and quantify chemically similar compounds based on relative signals. This approach is limited by the availability of heavy standards and, therefore, is effective only in targeted studies or within compound classes.

In absence of heavy standards such as in untargeted metabolomics or label-free quantification in proteomics, it remains challenging to ensure reproducibility such that it would be possible to compare samples from different experiments. A steadily growing arsenal of normalization methods allows correcting for differences in feature intensities across different batches (Chawade et al., 2014; Välikangas et al., 2018; Fu et al., 2022). However, they only tackle one facet of the reproducibility challenge. They are, however, ineffective in the case features could not be detected or matched across batches. This problem is quite frequent as caused by a multitude of common issues: drifts in retention times, loss of sensitivity, differences in tuning, changes in the matrix, contaminations, etc. Such irreproducible behavior cannot be corrected by posterior data processing. This has led to the development of approaches for testing LC-MS instrument performance *a priori*. Samples are injected only after the test is passed. Testing relies on three pillars: standard quality control (QC) samples, a standard acquisition method, and software to discover deviations (Broadhurst et al., 2018; Dogu et al., 2019; Kuhring et al., 2020) from expectations (Dogu et al., 2017; Kuhring et al., 2020). The importance of QC-based monitoring in metabolomics has been known for >15 years (Gika et al., 2007). However, little progress has been made in the past decade. The effort toward reproducible untargeted metabolomics is currently spearheaded by the metabolomics quality assurance and quality control consortium (mQACC (Beger and au, 2020; Evans et al., 2021)). A recent survey revealed that expert metabolomics labs employ procedures for system suitability testing (Evans et al., 2021) but the examples of literature discussing specific details or presenting solutions are very scarce (Broadhurst et al., 2018).

Here we propose means for systematic quality control (QC) and monitoring of instrument performance and properties for high-resolution mass spectrometry, focusing on a time-of-flight (TOF) instrument. We have been using TOF-MS productively for more than 10 years and analyzed more than 1 million samples by untargeted metabolomics. On occasions, we had to reanalyze entire batches of samples because of major problems and biases that passed unnoticed during instrument tuning. Hence, this work originated from the need to verify system suitability before injecting precious samples, and to monitor performance drifts that might require user intervention. Our system includes a chemically defined QC sample, a short acquisition method, software for extracting detailed spectral information from profile data, and a simple visualization service for end users.

## Results

We set out to implement a system suitability testing platform capable of quick, quantitative and comprehensive characterization of the state of a high-resolution ESI-TOF-MS instrument (Agilent 6550 iFunnel Q-TOF). On purpose, we omitted chromatography from testing. This decision was motivated by several practical reasons. Chromatography and detection are separated processes, and we frequently switch liquid chromatography systems depending on the type of separation needed (e.g., reversed phase, HILIC, ion-pairing). Chromatographic performance can be evaluated by means of retention time stability, height equivalent to a theoretical plate (HETP), peak tailing, etc., with samples that vary for the different methods. Here, we wanted to define a MS system suitability that is independent of a specific chromatographic setup and therefore more generally applicable. It was tailored to capture more information that relates to ionization, ion transmission and detection. We focused on negative mode ionization, which is predominant for metabolome profiling and less prone to adduct formation. The platform is composed of three core elements: a chemically defined quality control mix, an acquisition method, and a processing engine that extracts quantitative information from measured spectra to do analytics and reporting through a web service.

### Quality control mix

The first element is a chemically defined quality control (QC) sample. This should include analytes that allow testing the system, be stable over long periods, and ready to inject. For the purpose of testing an ESI-MS system, the QC analytes should span over the full mass range of interest (m/z 100 to 800 for us), be diverse in chemical properties (e.g., polarity, pKa), and in propensity of analytes to build adducts or fragment during ionization. Following these principles, we opted for a mix of nine compounds. We emphasize that there is likely ample room to further optimize the composition of the QC mix. In the current composition, it has been in use for almost 2 years with satisfactory results and, therefore, we have not tested different mixes. For each compound, the concentration was adjusted to obtain an intense monoisotopic peak. Typically, the nine analytes also produced 27 isotopic peaks, 1 adduct, and 9 fragments, for a total of 37 expected spectral peaks (Supplementary Table S1). During processing, the expected peaks are analyzed individually to quantify peak and ionization properties. Any additional peak that is detectable but not part of the expected set is considered background. All background peaks are treated as a collective to quantify purity and dirt of the system.

### Acquisition method

**FIGURE 1:**
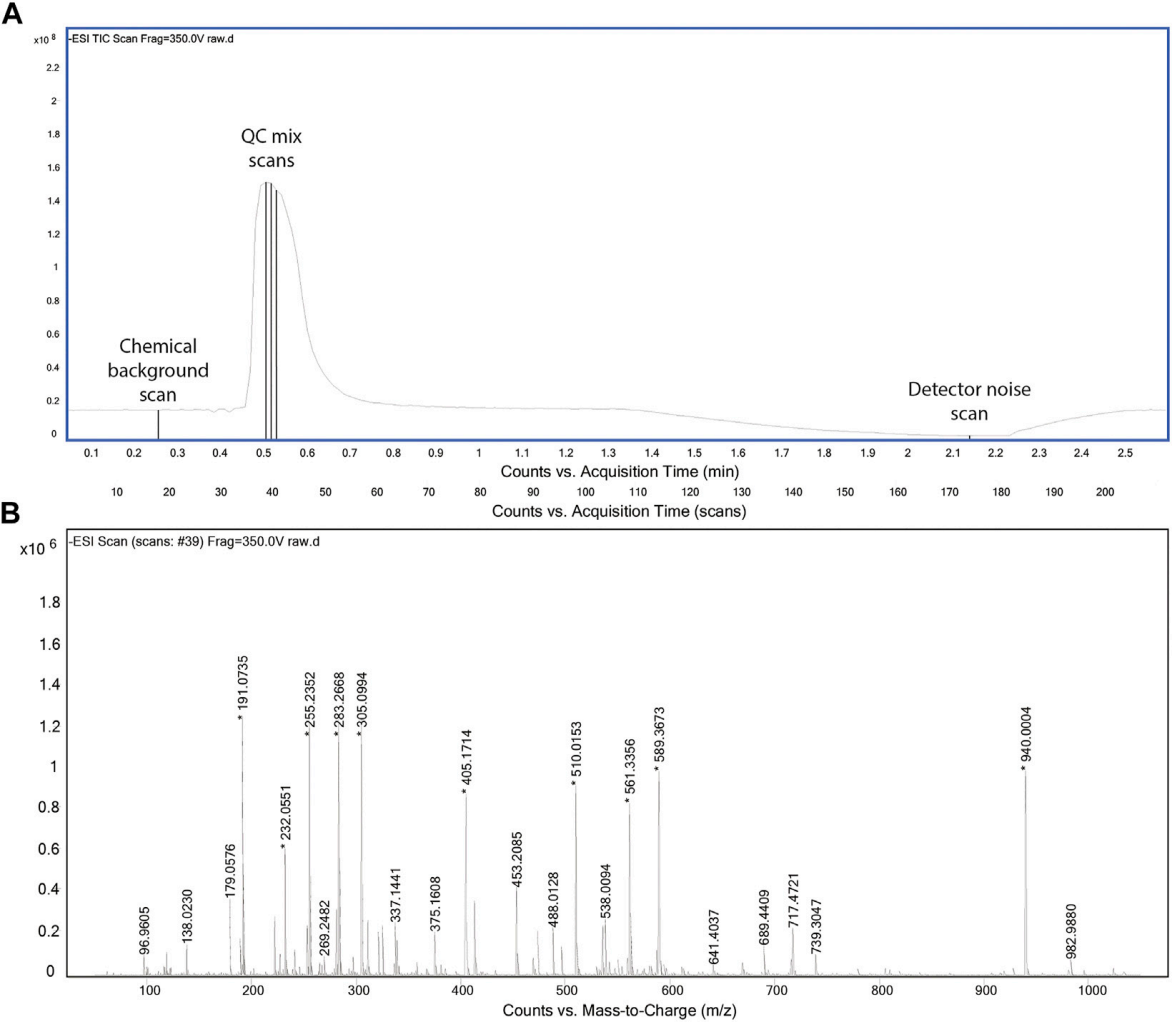
Total ion chromatogram of the acquisition method **(A)**. Representative spectrum for QC mix scans **(B)**.

The acquisition method was designed to collect critical data in possibly short time. For the aforementioned reasons, we omitted a chromatographic separation and used an instrument method similar to flow injection analysis with a solvent flow of 150 μL/min. In principle, we were interested in capturing three types of scans: 1) full spectra for the chemical (solvent) background, 2) full spectra for the QC mix, and 3) full spectra in the absence of ionization. The latter was included to potentially assess background noise of the detector. It was obtained by stopping the liquid flow to the source and recording residual ion counts. The final profile is shown in Figure 1. The chemical background is acquired first to fully reflect the equilibrated LC-MS system and to avoid being affected by any tail of the QC mix peak. Given the absence of a column between autosampler and ionization chamber, the injection program was modified to introduce a temporal delay of about 25 s between the start of MS acquisition and the injection of the QC mix. After the sample has cleared from the ionization chamber (about 80 s), the flow is stopped to acquire detector background. Finally, the flow is ramped again. Throughout the period of 2 min, the MS acquires full scan profile data in negative ionization mode at a frequency of 4 GHz.

### Feature extraction

Upon acquisition, raw profile data is analyzed to extract quantitative information that describes spectral properties in much depth. Not knowing in advance which properties of a spectrum drift or shift over time, we designed a very inclusive analysis that extracts 2,850 quantitative features for each QC sample injected. Ultimately, this information is obtained from a detailed analysis of five scans. The largest fraction of features is extracted from the chronogram peak related to the QC mix. The exact scan number is determined dynamically by picking the scan with the highest total ion current, and the analysis is extended to the two following scans to obtain an average value and a measure of deviation for each feature. For each of the 37 expected peaks of the QC mix, we collect intensity, absolute mass accuracy, factual ppm, multiple widths, area under peak tails, symmetry, goodness-of-fit with a Gaussian, number of subsequent peaks and their intensity ratios (Supplementary Figure S1).

For each expected isotopologue, fragment, or adduct, we also measure the height relative to the deprotonated, monoisotopic peak, as well as the difference to the theoretical relative abundance. This allows diagnosing deviations from linear response, excessive in-source fragmentation, or increased salt contaminations, respectively. For each of the above features, we record the mean value and the standard deviation from three consecutive scans. Overall, the numeric features derived from the QC mix peaks are 1720.

To capture baseline properties, level and type of dirt, instead of focusing on a predefined list of m/z features, we segmented the mass axis in windows of 50 amu. For each window, we recorded number of peaks, intensity sum, intensity percentiles with all expected ions excluded, intensities of 10 most abundant peaks, as well as their intensity percentiles. This resulted in 720 more features, so the total of features coming from the QC mix is 2,440.

Two other scans are analyzed to gain additional information about the system. The chemical background is characterized in a single scan before the QC mix peak, i.e., scan number 18 in our LC-MS setup. Similar to the QC mix scans, we extract 140 features related to two reference compounds that are co-sprayed and used as lock masses for intra-scan mass calibration (HOT and HEX in Supplementary Table S1). 180 more features come from the windows of 100 amu, making a total of 320 background-related features. Finally, pure detector signal is characterized in a late scan in absence of ionization. We collect 90 more features from the windows of 200 amu for a grand total of 2,850 for each injected QC sample.

Feature extraction was implemented in Python. In our environment, it is triggered automatically by the appearance of a QC acquisition file in a predefined location. The results are stored in a SQL database that constitutes the access point for all downstream analyses. Starting from raw MS profile data, our extracts all features and updates the corresponding logs and databases within 20 s. Including measurement, data logistics and web service rendering time, the full process takes less than 5 min. In the following, we showcase and discuss the immediate and long-term benefits of using the SST platform.

### Instrument monitoring

A primary goal of the aforementioned procedure is to verify system suitability before proceeding with data acquisition. For this purpose, we implemented a monitoring system to visualize and compare current and historical data. It consists of a web service that pulls data in real-time from the database with extracted QC features and reports key information on a dashboard. For obvious reasons, including all 2,850 features would have been problematic and inefficient. To favor visualization of accessible information over an overflow of data, we defined 16 quality indicators that report aspects of analytical relevance such as resolution, mass accuracy, accuracy of isotopic ratios, adduct formation, signal intensity, signal-to-noise, levels of dirt and detector noise. These indicators (Supplementary Table S2) were calculated from the 2,850 primary features and are presented for users on the dashboard.

Visualization was designed to inform on two types of patterns. First, we were interested in capturing particularly abnormal values of any of the quality indicators. Therefore, we integrated plots that visualize the distribution of quality indicator values in the past and the latest to be evaluated. To facilitate the analysis, the system also performs automatic outlier detection by the isolation forest algorithm (Liu et al., 2008). The latter is based on an ensemble of decision trees, followed by a correction routine specific to the type of the indicator. In real-time, each QC sample is scored automatically by counting the number of outliers across the 16 quality indicators. As a rule of thumb, if more than 4 values are classified as outliers, the QC sample flagged with bad quality.

The second type of pattern that we wanted to highlight is temporal trends. We hypothesized that factors such as detector aging or dirt accumulation could result in a subtle but continuous decay of instrument performance. Such drifts are slow and, therefore, they would not be recognized by outlier detection. We integrated a trend detection that uses linear models with empirical thresholds for R^2^ and slope coefficients. Three temporal intervals are considered (2 weeks, 1 month, and 2 months) and reported on the dashboard (Supplementary Figure S2).

To assess the technical reproducibility of feature extraction, we analyzed 191 QC samples acquired on the same day without any modification of instrument parameters, i.e., tuning. The median intra-day coefficient of variation across the 2,850 QC features was 27%, but with strong differences between the types of QC features. For example, noisier features were associated to the lock masses included in the buffer. Other noisy features described the tails of DC mix ions (i.e., ringing and baseline artefacts). For the 16 quality indicators, the median variation coefficient was 4%. This indicates that the setup is robust enough to capture shifts of about 10% or more.

The system has been operating in a pilot period of 21 months. During this period, at least one QC sample has been analyzed on 110 days constituting a total of 153 measurements. Among those, 37 QC samples featured four or more outlier values and were flagged as of bad quality. Based on automated outlier detection, *fragmentation_305* was found to be most out-of-order QC indicator (in 31% of QC samples, Supplementary Table S3), followed by *isotopic_presence* (28%) and *baseline_25_150* (25%). The most stable indicators were *average_accuracy* (only 8% of bad quality), *resolution_700* (6%) and *baseline_50_650* (5%). During the same pilot period, a total of 40 trends were automatically detected for the QC indicators within 2-month windows. For instance, an increasing *chemical_dirt* trend was detected between October and December 2019 (R^2^ = 0.7071, *n* = 16) and a decreasing *resolution_700* trend was detected between October and December 2020 (R^2^ = 0.6873, *n* = 14). Other examples of trend detection are given on Supplementary Figure S3.

Upon detection of a bad quality QC sample, the user was prompted to take corrective actions to restore normal range. Thus far, corrective actions were suggested based on expert knowledge and the kind of outlier. For example, in the case of a loss of, e.g., resolution, mass accuracy, or ion transmission, the system was retuned focusing on the relevant section of the optics or opting for a general system tune in more extreme cases. In the case of increases of, e.g., chemical background signals, the primary response was to purge the system, replace buffers, or clean the source and front optics. In the case of a drop in signal or signal-to-noise, we evaluated whether sensitivity of the detector (i.e., the voltage of the multichannel plate detector, or the amp gain) had to be readjusted. These recommendations were adopted by users to prevent the injection of samples before normal operation was verified.

### Analysis of QC features

**FIGURE 2:**
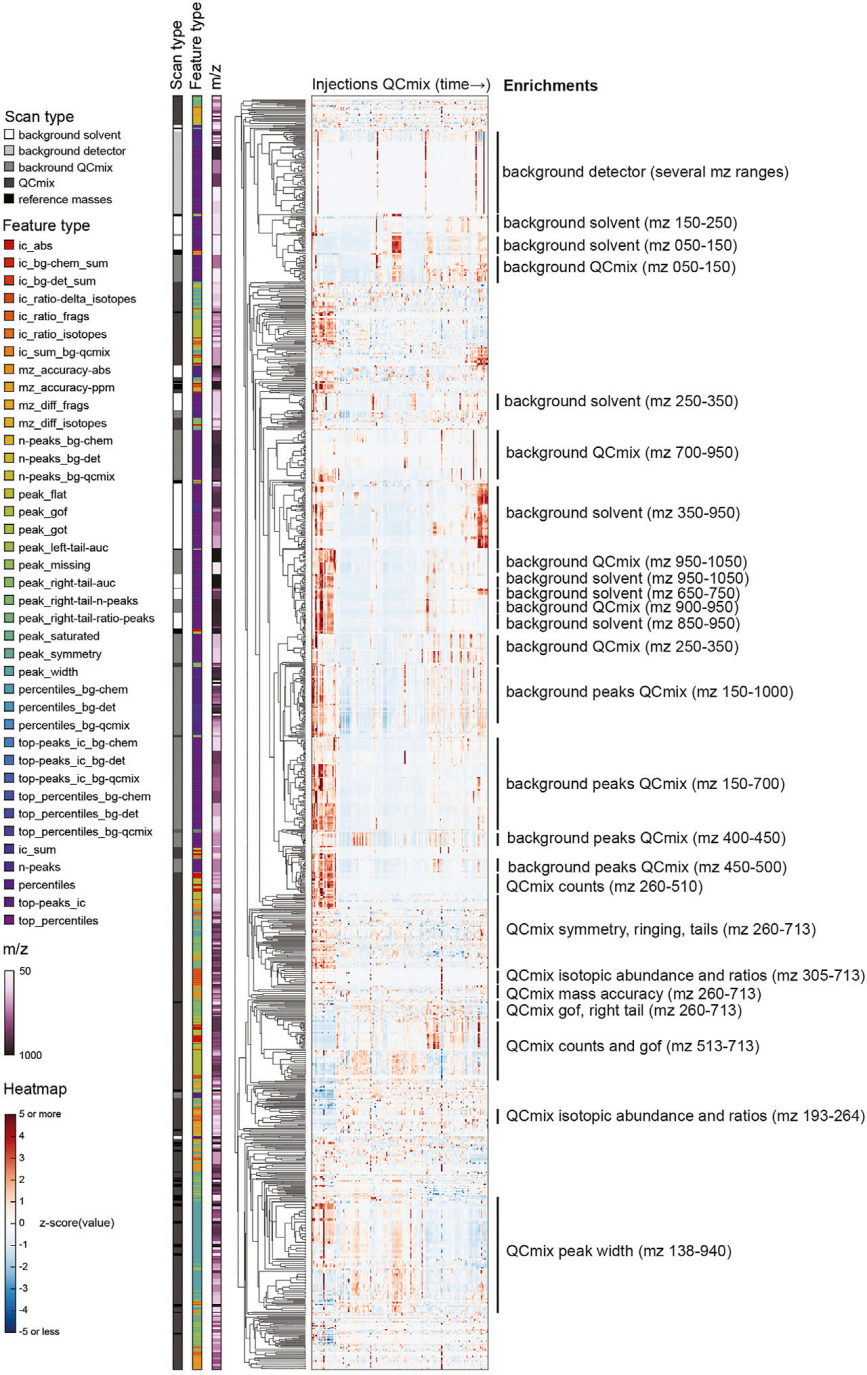
Hierarchical clustering of z-scored QC features with annotations by m/z, scan and feature types. See descriptions of feature types in Supplementary Tables S4, S5.

We designed the feature extraction to be very inclusive and capture possibly granular information on spectral properties and, in turn, instrument characteristics. This resulted in a long vector with 2,850 numerical values. By design, several of the features are likely correlated because they reflect similar aspects of the same peak, report the same property of different peaks of the QC mix, or simply relate to neighboring regions of the mass range. We therefore wondered about the actual information content: is there a benefit in collecting very detailed information or could one describe instrument state with much fewer features? To address this question, we analyzed the full matrix of values obtained in the initial 21 months of operations. We performed a principal component analysis (PCA) of the feature matrix and calculated a cumulative portion of total variance explained. We found that the first 10 components explained only about 52% of total variance. To capture 95%, 100 components were necessary. This highlights a substantial heterogeneity of the dataset. Rather than surfing through many principal components, we performed a hierarchical clustering to understand whether the list of QC features could be compressed without tangible losses in information (Figure 2). In line with the PCA, numerous subtrees with peculiar patterns over the measured QC samples emerged. To verify what kind of QC features correlated in these subtrees, we sought for enrichments in either the m/z range of the feature, its type, or the scan the feature was extracted from (encoded in the colors on the left of Figure 2).

Tightest clustering was found for features linked to spectral background (labeled with “background” in Figure 2), pointing to some redundancy. These are the values that are not related to the chemicals spiked in the QC mix. They dominated the upper part of the clustered heatmap. Background features of similar type but different mass range were frequently adjacent. Background features extracted from the solvent and from the QC mix scans co-clustered frequently. Retrospectively, this was expected because apart from the regions populated by QC mix ions, the two scans are expected to be identical. In such cases, a single feature measured in a QC mix scan seems sufficient to recapitulate the principal drifts and shifts observed across scans and the mass range. In contrast, the features derived from ions deriving from the chemicals spiked in the QC mix were more heterogeneous (lower part of Figure 2). Albeit many features of similar type were close in the tree (e.g., symmetry, isotopic properties, mass accuracy, etc.), their distance was generally higher than observed for background features. This seems to reflect the fact that peak features tend to vary across the mass range, possibly because of differences in intensity or in the spectral neighborhood. The only exception was the peak width, which correlated well across the whole mass range.

To further investigate feature redundancy, we did a cross-correlation analysis between all 2,417 continuous QC features. The resulting Pearson correlation coefficients were <0.5 in 95% of the pairs. Only for 0.4%, the correlation was strong ( |r| > 0.9). As expected, these cases were related either to the features of the same type or to “synonymic” features of the same m/z window. These results confirm that QC features describe many spectral properties, not likely to be fungible. It is further supported by a simple visual analysis of the differences between injections, i.e., the columns on the heatmap shown in Figure 2. Many vertical stripes emerge, which indicate sets of features with values at the far ends of the measured range. Importantly, the extreme values are not aligned vertically over a large fraction of features but tend to vary across samples. This heterogeneity indicates that fine-grained shifts are present in the data, even though they might have not been captured by the 16 quality indicators that were adopted for instrument monitoring. It remains to be tested if such drifts had a tangible effect on the measurement of studies that were acquired on the same day. This analysis would require expanding the outlier detection introduced for system monitoring to all measurable features. Whenever an extreme deviation was reported, a set of test samples ought to be run to evaluate the practical consequences.

### Association analysis between instrument settings and QC features

We were wondering whether any of the aberrant behaviors detected during the pilot phase were associated to drifts in setpoints or readbacks of the instrument. Therefore, we studied the relationship between measured quality indicators (or QC features) and actual instrument settings, which include both tunable and non-tunable values. Tunable values affect ion optics and detection and are adjusted during instrument tuning or calibration according to the procedures that are implemented in the control software. Non-tunable values consist of readbacks of parameters such as pressures, currents, pump speeds, noise, and are collected for diagnostic purposes. In many cases, they are stored with each run or in the tuning reports. The number and type of accessible instrument settings varies across vendors and type of instruments. For the QTOF instrument described here, about ten non-tunable values and fifty tunable values were extracted for each QC run and stored.

**FIGURE 3:**
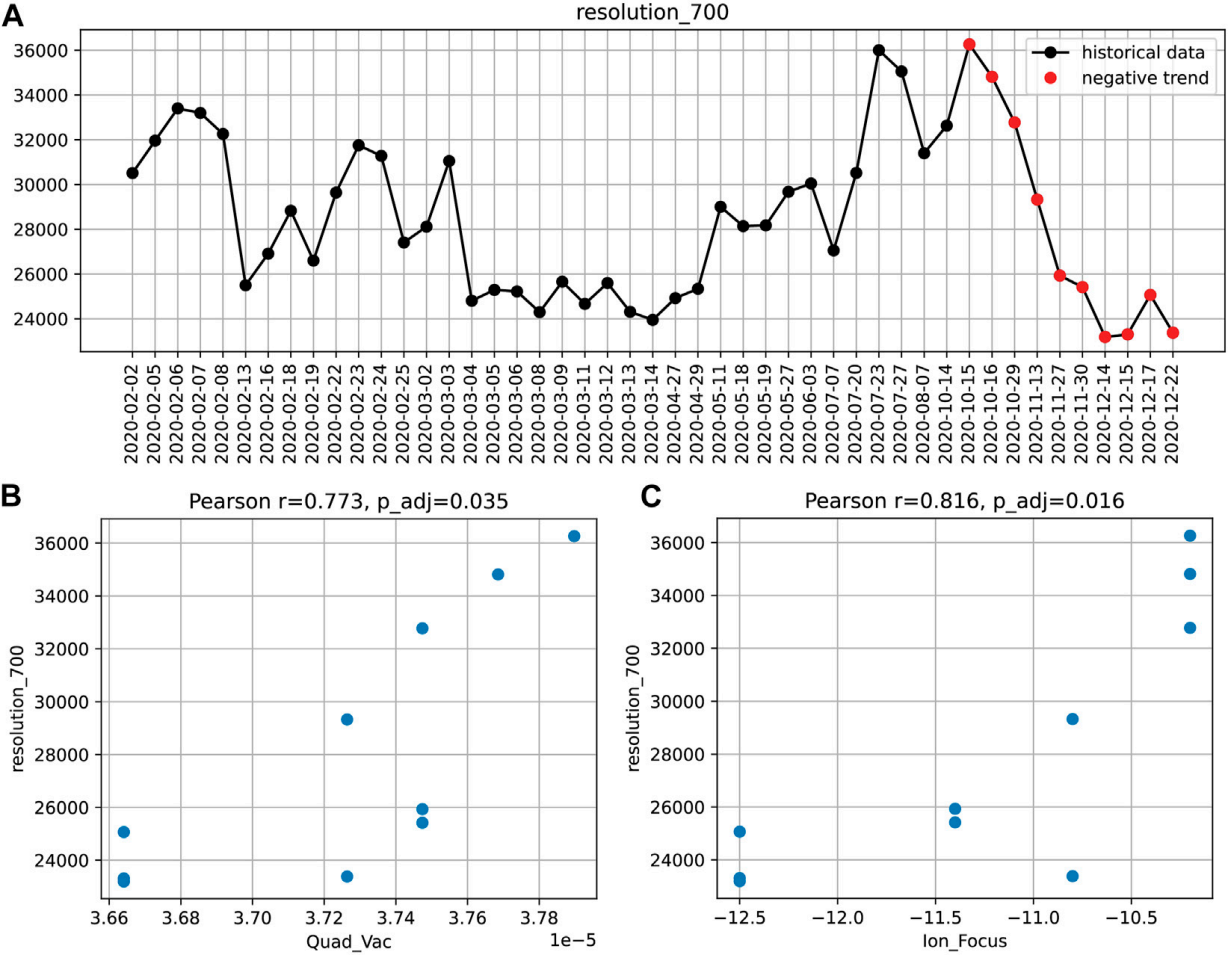
The decreasing *resolution_700* trend **(A)** and the corresponding correlations with the instruments settings: quadrupole vacuum **(B)** and ion focus **(C)**. Pearson correlation coefficients and Bonferroni adjusted *p*-values are given.

The connection between instrument performance and instrument settings can be analyzed by different approaches. For example, a significant decay in resolution (at m/z 700) over the period of 2 months was once identified by the aforementioned trend detection (Figure 3).

To find potential causes of this loss in performance, we sought for correlations with instrument settings during the same period. Significant associations were found for two parameters: the non-tunable pressure reading in the first quadrupole and the ion focus strength (Figure 3). This suggests that the transient drop in resolution might be caused by an increased spread of trajectories or velocities of ions in the section preceding the TOF pulser.

An alternative approach is to seek for parameters that are associated with top performance. We illustrate this for the signal-to-noise, which reflects instrument sensitivity. In this case, we split all QC samples into groups of high (top 20th percentile) and low signal-to-noise. We then sought for instrument settings that were different between the two groups. The procedure was repeated for another quality indicator, the signal-to-background (Table 1). For both indicators, we found significant associations. Several expected associations were found. For instance, better vacuum (lower value of *TOF_Vac*) was linked to higher signal-to-noise and signal-to-background. Increased multichannel plate voltage (*MCP*), or instrument firmware (*InstrumentFW*) improved both indicators. We also found less intuitive associations, like the voltage of several lenses of the ion optics before the TOF section (e.g., *Top_Slit, Bottom_Slit, Lens_2*, *Oct_1_RF_Vpp_1*, etc.) which are likely to overall improve ion transmission.

**TABLE 1:** Statistical comparison of the machine tunes. Machine tunes significantly different between signal-to-noise groups are shown on the left. Machine tunes significantly different between signal-to-background groups are shown on the right. In both cases, three statistical tests were applied for each comparison (Kolmogorov-Smirnov, Mann-Whitney U, Kruskall), followed by FDR correction for multiple testing. The biggest significant *p*-value is reported. Sign of linear relationship is shown, where the medians of two distributions were different.

Signal-to-noise			Signal-to-background		
Setting	Sign	*p*-value	Setting	Sign	*p*-value
*Acc_Focus*	—	0.0118	*Amp_Offset*		0.0044
*Amp_Offset*		<0.0001	*InstrumentFW*	+	0.0414
*Bot_Slit*	+	0.0229	*Length_of_Transients*		0.0443
*Cell_Entr*	+	0.0433	*Lens_2*		0.0414
*Cell_Exit*		0.0206	*Lens_2_RF_Ph*		0.0255
*InstrumentFW*	+	0.0001	*MCP*		0.0034
*Lens_2*		0.0007	*Oct_1_RF_Vpp_1*		0.0003
*Lens_2_RF_Ph*		0.0007	*Puller_Offset*		0.0044
*MCP*	+	0.0001	*TOF_Vac*	—	0.0091
*Mirror_Mid*	+	0.0156	*Top_Slit*	+	0.0044
*Oct_1_RF_Vpp_1*		<0.0001			
*Puller_Offset*		0.0001			
*TOF_Vac*	—	0.0003			
*Top_Slit*	+	<0.0001			

**FIGURE 4:**
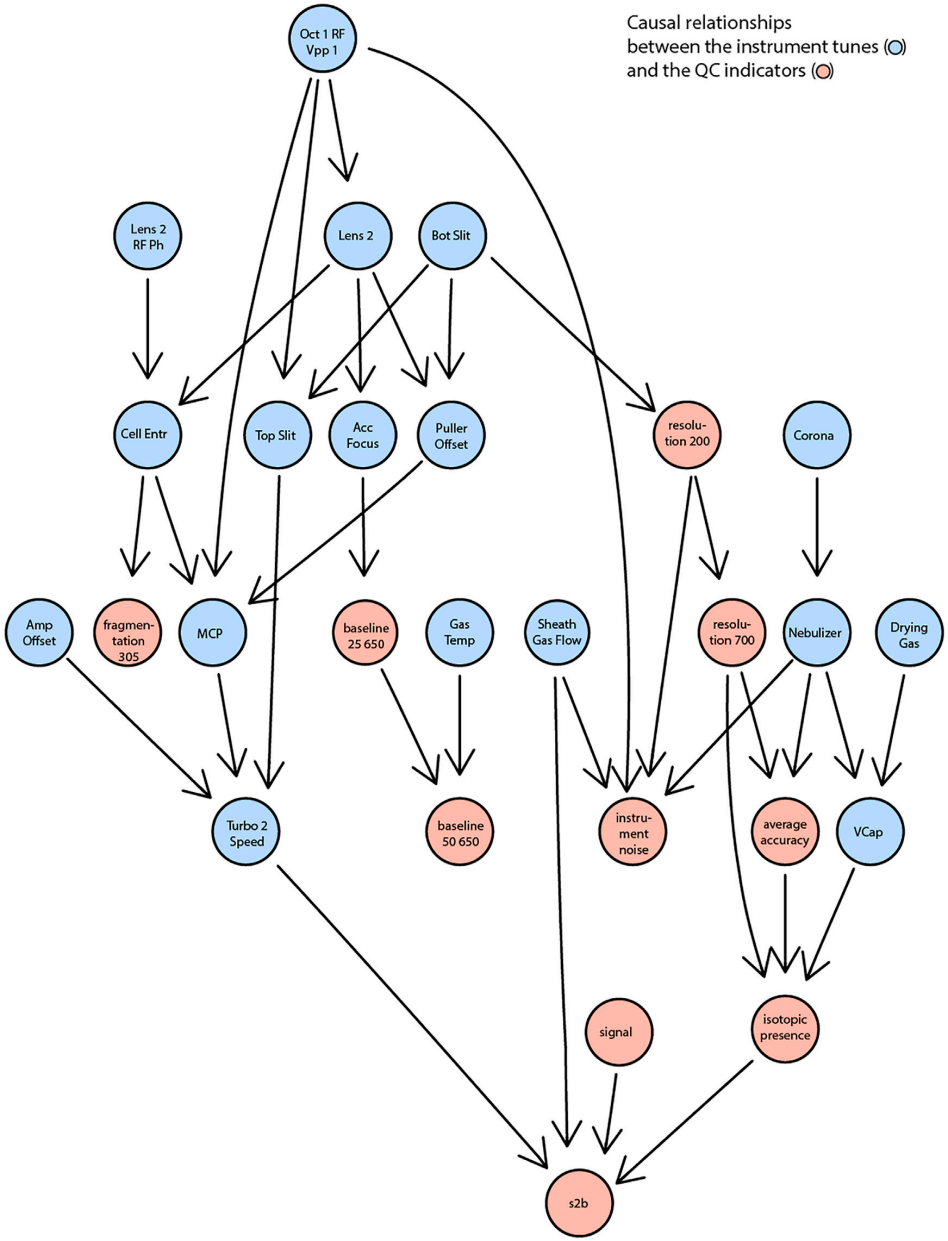
A reduced DAG reflecting the causal relationships between the instrument settings (in blue) and the QC indicators (in red). A significance level of 0.15 was used as the threshold for conditional independence testing.

All identified associations may indicate what settings determine properties of measured spectra but were analyzed in isolation and for selected examples. In reality, settings and indicators are partly interdependent. For example, adjusting a voltage to increase sensitivity might negatively affect resolution. To go beyond individual correlations and statistical tests, we attempted to learn causal relationships between the tunable instrument settings and the observed quality indicators. Specifically, we attempted to learn the underlying structure from all available data using the PC (Kalisch et al., 2012; Kalisch et al., 2020) algorithm (named after Peter and Clark (Spirtes et al., 1993)) and conditional independence testing. The result is a directed acyclic graph that condenses statistical dependence between instrument settings and performance indicators (Figure 4). The results reveal that, for example, the spontaneous fragmentation of fluconazole (*fragmentation_305*) was associated to voltages in the section that precedes the collision cell (*Lens_2, Lens_2_RF_Ph, Oct_1_RD_Vpp_1, Cell_Entr*). Resolution (both at m/z 200 and 700) seems governed by the bottom slicer voltage. This is coherent with the function of the slicer, which flattens the ion beam before it is pulsed orthogonally in the flight tube. Suboptimal slicer settings increase differences in the length of the flight path which would result in different flight times even for ions of identical m/z. Thereby, it would worsen peak resolution. Instead, most settings related to the electrospray ionization process (aggregated on the right part of Figure 4) affect parameters related to signal intensity such as signal-to-baseline, isotopic accuracy, mass accuracy. With more data, it could be possible to build a reliable statistical model to also infer which settings should be adjusted to achieve or maintain a certain property of the measurement.

## Conclusion

We present the concept of a system suitability testing platform for monitoring the status of a high-resolution QTOF mass spectrometer. The setup consists of a QC mixture, an acquisition method, software to extract a detailed ensemble of quantitative features describing spectral properties, and a simple R Shiny front-end for real-time visualization. We operated the testing platform in a pilot lasting for 21 months and including 153 individual measurements of the QC mixture. We demonstrated instrument monitoring by a small set of quality indicators (16 in our case) and the implementation of routines for trend and outlier detection. The platform, therefore, helps users in evaluating in depth the instrument readiness to measure biological samples. In most cases, unsatisfactory results could be effectively addressed by cleaning the ion optics or a thorough tuning/calibration of the instrument.

The presented setup offers ample room for further improvements. In particular, the long-term stability of the QC mixture should be verified. The feature set could also be optimized. The feature extraction is generic and easily transferrable to TOF instruments from other vendors. Adaptation to high-resolution instruments that use a Fourier Transform to reconstruct spectra would require more work. Features related to peak symmetry, detector ringing, baseline shifts, etc. are irrelevant. In contrast, it would be important to include features that can capture artifacts of FT spectra: harmonic peaks, coalescence, etc.

Further, we illustrated how collection of instrument setpoints and readbacks allows to derive the causal relationships between instrument settings and instrument performance measured with the QC mixture. This highlights additional, potential applications of the testing platform. First, we envisage that the system could recommend instrument settings to maintain or attain a particular value of a quality indicator. Second, it could assist in timing preventive maintenance. We speculate that continuous QC data collection coupled with predictive models would be able to indicate when to replace wearable parts, clean specific parts of the ion path, or maybe even anticipate major failures such as a pump breakdown.

Thus far, the testing platform has been conceived to operate with data pertaining to a single instrument. Future work will explore the possibility of using the detailed information provided by the QC mix to harmonize data collected either on the same instrument but at different time points, or on different instruments of the same type. The canonical approach to normalize across batches or instruments is to include internal standards, or standard reference materials. This approach, however, works only for compounds that are present in the standard material and fails to capture non-linear effects. We hypothesize that capturing detailed information on, e.g., baseline, ion transmission, fragmentation, etc. might help to harmonize data before normalization by standards can be applied.

## Methods

### Instrument details

All analyses were done on an Agilent 6550 Q-TOF instrument, operated in negative ionization and 4 GHz High-Resolution mode because it matches the configuration that we use in routine flow injection and LC-MS analysis. The mobile phase was 60% isopropanol in water (v/v) supplemented with homotaurine (Sigma-Aldrich, Germany) and Hexakis (1H, 1H, 3H-tetrafluoropropoxy) phosphazine (Agilent) as reference masses for m/z axis calibration. The solvent flow was 150 μL/min. All compounds included in the QC mixture (Supplementary Table S1) were purchased from Sigma-Aldrich (Germany) at the highest purity available. The injection volume was 1 μl.

### Anomaly detection and quality control

We implemented two types of anomaly detection: 1) based on descriptive statistics, and 2) based on machine learning. Both approaches require some reference (or training) data to apply algorithms and determine whether a new quality indicator is likely to be an outlier or not. Each of the 16 quality indicators of the new run is evaluated individually, and the total number of non-outliers serves as the QC run score. Two different types of anomaly detection suggest different usage scenarios of the monitoring system.

The statistical approach assumes that the instrument preserves its properties within the period of the study. If the instrument performance remains the same with only little oscillations, appropriately, any quality indicator does as well. Thus, measuring quality indicators repeatedly over time makes it possible to derive confidence intervals for the expected mean, or the ranges that are considered as “good” or “bad” for each indicator. In this approach, we use quantiles to compute such ranges, as soon as enough data is generated and stored in the database. We set 60 measurements of the QC sample to be enough to classify further values of quality indicators as “good” or “bad,” i.e., within the expected interval or not. This number, however, is only empirical and remains a configurable parameter in the platform.

This approach may not be optimal for longitudinal studies, because it does not adapt to the changes in the instrument state over time. Intervals derived for the first *N* measurements will be applied to the data forever. Possible effects of instrument aging and hardware replacements will be ignored. To potentially account for them and to make the system adaptable, we implemented another approach based on machine learning. Isolation Forest, an unsupervised method for outlier detection, is used to re-evaluate all the entries in the database as soon as a new QC measurement is acquired. This way, the platform adapts to the gradual temporal drifts in quality indicators, while still being capable of detecting anomalies. Because of that, only *N =* 20 measurements are set as a minimum number of entries to apply the method.

Both methods’ predictions are corrected for the type of the quality indicator. For instance, low mass accuracy values are not treated as outliers, since a small difference between expected and measured m/z value for an ion is desired. Big signal-to-noise ratios are not treated as outliers, since high signal-to-noise ratio is preferable, in general. For other cases, adjustments are made on top of the aforementioned methods to compensate for artifacts caused by little data available (i.e., when the total number of QC samples in the database is still small).

In our experience, both methods to detect anomalies have shown similar results, when applied to the data systematically acquired within 21 months. However, in multi-day acquisitions, we see Isolation Forest to be preferable due to its adaptability and relative robustness. Isolation Forest is, therefore, a default method in the platform.

### Web service

We used R Shiny framework to implement a web service providing users with graphical representation of the data and analytics (Supplementary Figure S2). The layout contains three tabs: *summary*, *trends* and *table* components. The *summary* tab allows users to select a QC run by date and to see how the corresponding quality indicators are aligned against the full dataset. Score of the selected run and the distributions for each metric are displayed. The *trends* tab depicts temporal progression for the selected indicator, marking overall run qualities. This allows to see and analyze each metric’s behavior retrospectively. Linear trends are computed and visualized as well, which helps detecting gradual loss of sensitivity, gain of dirt in the system, etc. Finally, the *table* tab explicitly shows the values of the quality indicators from the database. Values classified as “good” or “bad” are colored in green and red, respectively.

### Availability

The source code of the SST platform core (raw signal processing, feature extraction and engineering) is available at https://github.com/zamboni-lab/SST-platform-core. The QC database and the Shiny app of the web service are available at https://github.com/zamboni-lab/SST-platform-shiny.

## Data Availability

The datasets presented in this study can be found in the GitHub code repository at https://github.com/zamboni-lab/SST-platform-shiny/data/.
