# Supplementary material for: A system suitability testing platform for untargeted, high-resolution mass spectrometry
Source: Front Mol Biosci. 2022 Oct 11;9:1026184. doi: 10.3389/fmolb.2022.1026184 (PMC9592825; doi:10.3389/fmolb.2022.1026184)
Supplement: Supplementary file 1 [file Table1.DOCX]

**Table S1.** Expected ions of QC mix and buffer

|  | **QC mix ions** | | |  | **QC mix ions** | | | | |
| --- | --- | --- | --- | --- | --- | --- | --- | --- | --- |
| **#** | **compound** | **m/z** | **type** |  | **#** | **compound** | **m/z** | **type** |  |
| 1 | *Caffeine* | 193.0725 | isotope |  | 25 | *Perfluorodecanoic acid* | 512.9594 | isotope |  |
| 2 |  | 194.0759 | isotope |  | 26 |  | 513.9628 | isotope |  |
| 3 |  | 195.0792 | isotope |  | 27 |  | 514.9662 | isotope |  |
| 4 |  | 179.0569 | fragment |  | 28 |  | 468.9696 | fragment |  |
| 5 | *Fluconazole* | 305.0962 | isotope |  | 29 | *Tricosa-fluorododecanoic acid* | 612.9531 | isotope |  |
| 6 |  | 306.0995 | isotope |  | 30 |  | 613.9564 | isotope |  |
| 7 |  | 307.1029 | isotope |  | 31 |  | 614.9598 | isotope |  |
| 8 |  | 191.0681 | fragment |  | 32 |  | 568.9632 | fragment |  |
| 9 | *Albendazole* | 264.0806 | isotope |  | 33 | *Perfluorotetra-decanoic acid* | 712.9467 | isotope |  |
| 10 |  | 265.0840 | isotope |  | 34 |  | 713.9500 | isotope |  |
| 11 |  | 266.0873 | isotope |  | 35 |  | 714.9534 | isotope |  |
| 12 |  | 188.9996 | fragment |  | 36 |  | 668.9568 | fragment |  |
| 13 |  | 232.0544 | fragment |  | 37 |  | 646.9549 | fragment |  |
| 14 | *Triamcinolone acetonide* | 433.2026 | isotope |  |  | | | | |
| 15 |  | 434.2059 | isotope |  | **Buffer ions** | | | | |
| 16 |  | 435.2093 | isotope |  | **#** | **compound** | **m/z** | **type** |  |
| 17 |  | 453.2088 | adduct |  | 1 | *HOT (Homotaurine)* | 138.0224 | isotope |  |
| 18 |  | 337.1439 | fragment |  | 2 |  | 139.0258 | isotope |  |
| 19 | *Pentadeca-fluoroheptyl* | 368.9760 | isotope |  | 3 |  | 140.0291 | isotope |  |
| 20 |  | 369.9794 | isotope |  | 4 | *HEX (Hexakis (1H, 1H, 3H-tetrafluoro-propoxy) phosphazine)* | 940.0009 | isotope |  |
| 21 |  | 370.9827 | isotope |  | 5 |  | 941.0042 | isotope |  |
| 22 | *3-Heptadeca-fluorooctylaniline* | 510.0150 | isotope |  | 6 |  | 942.0076 | isotope |  |
| 23 |  | 511.0184 | isotope |  |  |  |  |  |  |
| 24 |  | 512.0217 | isotope |  |  |  |  |  |  |
