# Supplementary material for: A system suitability testing platform for untargeted, high-resolution mass spectrometry
Source: Front Mol Biosci. 2022 Oct 11;9:1026184. doi: 10.3389/fmolb.2022.1026184 (PMC9592825; doi:10.3389/fmolb.2022.1026184)

Figure S2. Snapshots of the web-service. A *summary* tab (top right): distributions of the quality indicators are displayed with red dotted lines indicating the selected run. A *trends* tab (left): historical data is displayed for the selected quality indicator on top. A table with summary on the trends and two trend plots are displayed below. A *table* tab (bottom right): values of the quality indicators are displayed in a table for the last 100 QC runs. Indicators classified as ‘good’ and ‘bad’ (read “within a normal range” and “likely an outlier”) are in green and red, respectively.

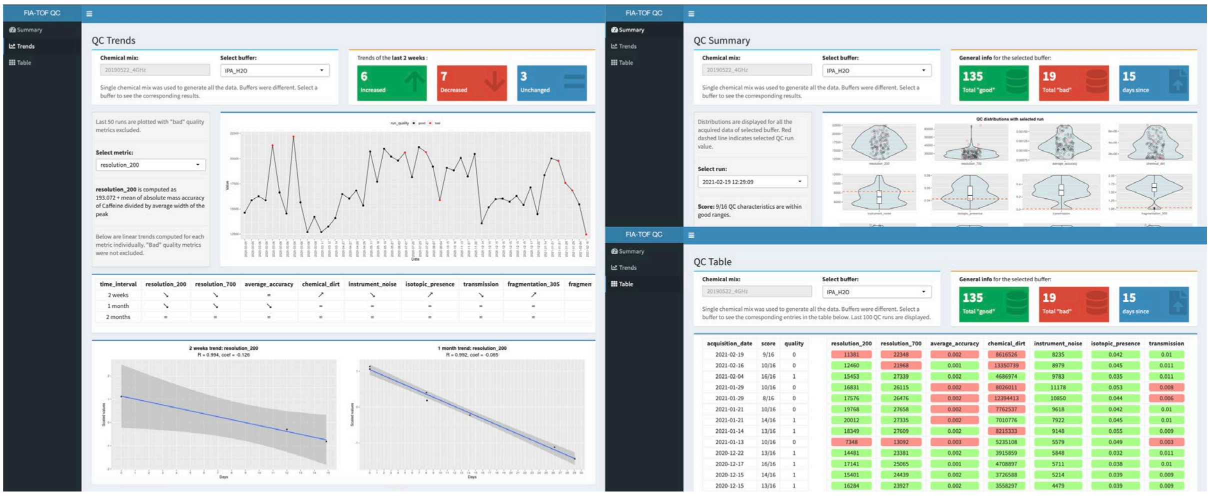

Supplement: Supplementary file 2 [file Image2.PDF]
