# Supplementary material for: A system suitability testing platform for untargeted, high-resolution mass spectrometry
Source: Front Mol Biosci. 2022 Oct 11;9:1026184. doi: 10.3389/fmolb.2022.1026184 (PMC9592825; doi:10.3389/fmolb.2022.1026184)

Figure S3. Examples of trend detection for quality indicators. Two-weeks trends are shown on top and one-month trends are below. Linear regression coefficient defines the sign of the trend, whereas the R^2 score reflects its significance. Empirical thresholds allow to classify the quality indicators as increasing, decreasing or unchanged (no significant trend detected) within the corresponding time period.

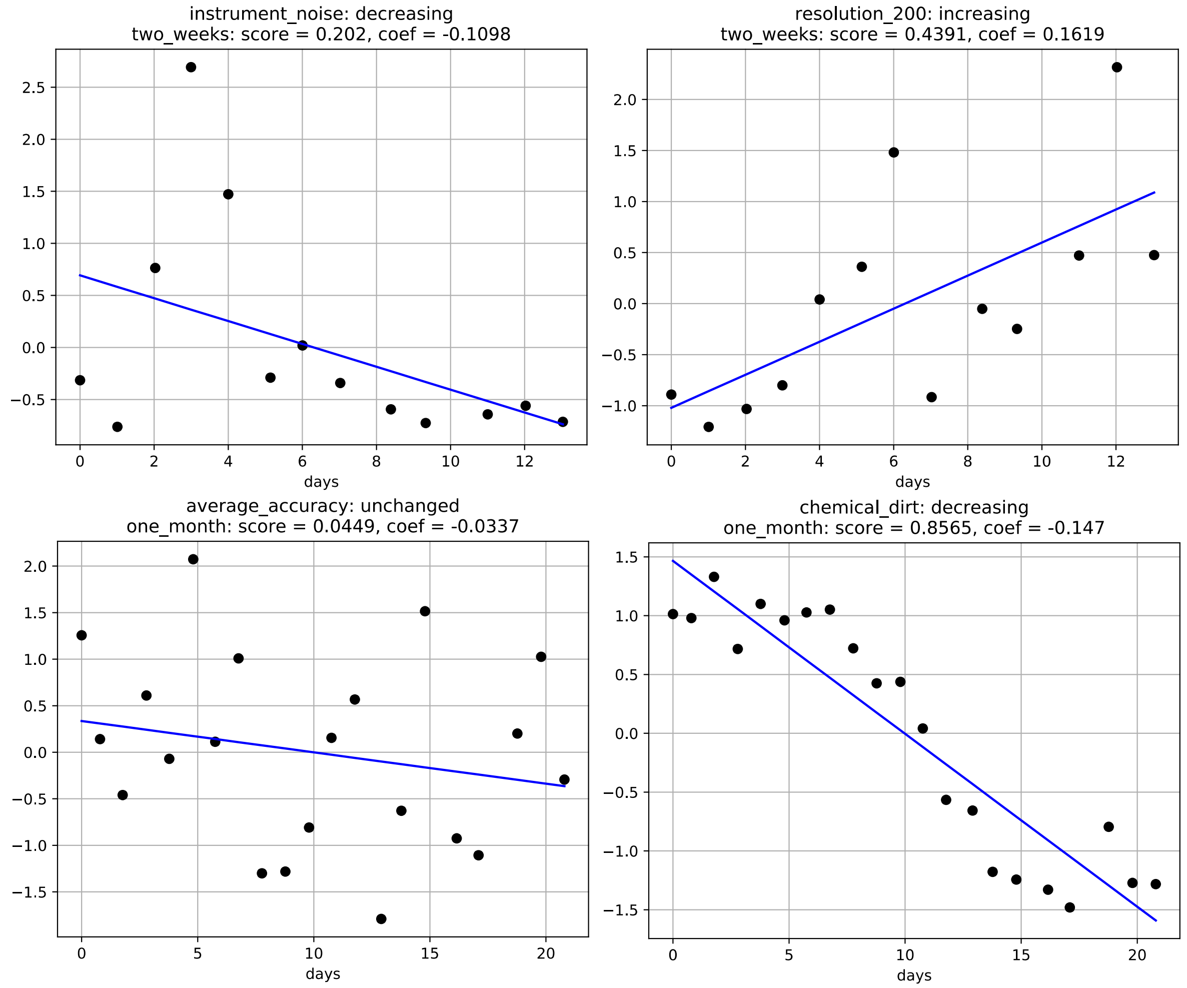

Supplement: Supplementary file 3 [file Image3.PDF]
