# Supplementary material for: A system suitability testing platform for untargeted, high-resolution mass spectrometry
Source: Front Mol Biosci. 2022 Oct 11;9:1026184. doi: 10.3389/fmolb.2022.1026184 (PMC9592825; doi:10.3389/fmolb.2022.1026184)
Supplement: Supplementary file 4 [file Table2.DOCX]

**Table S2.** QC indicators descriptions

| **QC indicators** | | | |
| --- | --- | --- | --- |
| **#** | **name** | **pseudo code** | **description** |
| 1 | *resolution_200* | mz_Caffeine / peak_width_Caffeine | measured m/z of Caffeine divided by the average width of the peak |
| 2 | *resolution_700* | mz_Perf_acid / peak_width_Perf_acid | measured m/z of Perfluorotetradecanoic acid divided by the average width of the peak |
| 3 | *average_accuracy* | **sum**(mean_abs_mass_accuracy_array) / n_ions | sum of mean absolute mass accuracy for all 37 ions divided by its number |
| 4 | *chemical_dirt* | **sum**(chem_bg_intensity_array) | sum of all intensities in the chemical background scan |
| 5 | *instument_noise* | **sum**(noise_intensity_array) | sum of all intensities in the detector noise scan |
| 6 | *isotopic_presence* | **sum**(**abs**(mean_iso_ratios_diffs_array)) / **length**(mean_iso_ratios_diffs_array) | sum of all isotope ratios’ diffs (in absolute numbers) divided by its number |
| 7 | *transmission* | mean_intensity_Perf_acid / mean_intensity_Fluconazole | mean of the Perfluorotetradecanoic acid intensity (m/z ~712) divided by the Fluconazole intensity (m/z ~305) |
| 8 | *fragmentation_305* | mean_intensity_Fluconazole_fragment / mean_intensity_Fluconazole | mean of Fluconazole fragment intensity (m/z ~191) divided by the Fluconazole intensity (m/z ~305) |
| 9 | *fragmentation_712* | mean_intensity_Perf_acid_fragment / mean_intensity_Perf_acid | mean of Perfluorotetradecanoic acid fragment intensity (m/z ~668) divided by the Perfluorotetradecanoic acid intensity (m/z ~712) |
| 10 | *baseline_25_150* | **percentile**(chem_bg_intensities_150_250, 25) | 25th intensity percentile from a [150, 250] m/z range of a chemical background scan |
| 11 | *baseline_50_150* | **median**(chem_bg_intensity_array_150_250) | median intensity from a [150, 250] m/z range of a chemical background scan |
| 12 | *baseline_25_650* | **percentile**(chem_bg_intensity_array_650_750, 25) | 25th intensity percentile from a [650, 750] m/z range of a chemical background scan |
| 13 | *baseline_50_650* | **median**(chem_bg_intensities_650_750) | median intensity from a [650, 750] m/z range of a chemical background scan |
| 14 | *signal* | **sum**(mean_intensity_array) | sum of mean intensities for all 37 ions |
| 15 | *s2b* | mean_intensity_3Hepta / **percentile**(intensity_array_500_550, 25) | mean intensity of 3-(Heptadecafluorooctyl)aniline (m/z ~510) divided by mean 25th intensity percentile from a [500, 550] m/z range |
| 16 | *s2n* | mean_intensity_3Hepta / (**median**(intensity_array_500_550) - **percentile**(intensity_array_500_550, 25)) | mean intensity of 3-(Heptadecafluorooctyl)aniline (m/z 510) divided by the diff between the median and the 25th intensity percentile from a [500, 550] m/z range |
