# Supplementary material for: A system suitability testing platform for untargeted, high-resolution mass spectrometry
Source: Front Mol Biosci. 2022 Oct 11;9:1026184. doi: 10.3389/fmolb.2022.1026184 (PMC9592825; doi:10.3389/fmolb.2022.1026184)
Supplement: Supplementary file 5 [file Table5.DOCX]

**Table S5.** Description of background feature types

| **Types of background features** | | | |
| --- | --- | --- | --- |
| **#** | **type** | **feature** | **description** |
| 1 | *background QCmix* | number_of_peaks_norm | a total number of peaks within a particular amu window of a QC mix scan with 37 expected ions excluded |
|  |  | intensity_sum_norm | an intensity sum of all peaks within a particular amu window of a QC mix scan with 37 expected ions excluded |
|  |  | percentiles_norm | 25th, 50th and 75th percentiles of intensities within a particular amu window of a QC mix scan with 37 expected ions excluded |
|  |  | top_peaks_intensities_norm | intensities of top 10 ion peaks within a particular amu window of a QC mix scan with 37 expected ions excluded |
|  |  | top_percentiles_norm | 25th, 50th and 75th percentiles of intensities of top 10 ion peaks within a particular amu window of a QC mix scan with 37 expected ions excluded |
| 2 | *background solvent* | number_of_peaks_chem | a total number of peaks within a particular amu window of a chemical background scan with 2 expected ions excluded |
|  |  | intensity_sum_chem | an intensity sum of all peaks within a particular amu window of a chemical background scan with 2 expected ions excluded |
|  |  | percentiles_chem | 25th, 50th and 75th percentiles of intensities within a particular amu window of a chemical background scan with 2 expected ions excluded |
|  |  | top_peaks_intensities_chem | intensities of top 10 ion peaks within a particular amu window of a chemical background scan with 2 expected ions excluded |
|  |  | top_percentiles_chem | 25th, 50th and 75th percentiles of intensities of top 10 ion peaks within a particular amu window of a chemical background scan with 2 expected ions excluded |
| 3 | *background detector* | number_of_peaks_bg | a total number of peaks within a particular amu window of a detector noise scan |
|  |  | intensity_sum_bg | an intensity sum of all peaks within a particular amu window of a detector noise scan |
|  |  | percentiles_bg | 25th, 50th and 75th percentiles of intensities within a particular amu window of a detector noise scan |
|  |  | top_peaks_intensities_bg | intensities of top 10 ion peaks within a particular amu window of a detector noise scan |
|  |  | top_percentiles_bg | 25th, 50th and 75th percentiles of intensities of top 10 ion peaks within a particular amu window of a detector noise scan |
