# Supplementary material for: A system suitability testing platform for untargeted, high-resolution mass spectrometry
Source: Front Mol Biosci. 2022 Oct 11;9:1026184. doi: 10.3389/fmolb.2022.1026184 (PMC9592825; doi:10.3389/fmolb.2022.1026184)
Supplement: Supplementary file 6 [file Table3.DOCX]

**Table S3.** Percentage of outliers detected for QC indicators

| **QC indicators** | | |
| --- | --- | --- |
| **#** | **name** | **percent** |
| **1** | *resolution_200* | 21 |
| **2** | *resolution_700* | 6 |
| **3** | *average_accuracy* | 8 |
| **4** | *chemical_dirt* | 21 |
| **5** | *instument_noise* | 13 |
| **6** | *isotopic_presence* | 28 |
| **7** | *transmission* | 21 |
| **8** | *fragmentation_305* | 31 |
| **9** | *fragmentation_712* | 24 |
| **10** | *baseline_25_150* | 25 |
| **11** | *baseline_50_150* | 23 |
| **12** | *baseline_25_650* | 6 |
| **13** | *baseline_50_650* | 5 |
| **14** | *signal* | 24 |
| **15** | *s2b* | 21 |
| **16** | *s2n* | 17 |
