# Supplementary material for: A system suitability testing platform for untargeted, high-resolution mass spectrometry
Source: Front Mol Biosci. 2022 Oct 11;9:1026184. doi: 10.3389/fmolb.2022.1026184 (PMC9592825; doi:10.3389/fmolb.2022.1026184)
Supplement: Supplementary file 7 [file Table4.DOCX]

**Table S4.** Description of QC mixture feature types

| **Types of QC mix features** | | | |
| --- | --- | --- | --- |
| **#** | **type** | **feature** | **description** |
| 1 | *counts* | intensity | measured intensity of the ion |
| 2 | *peak width* | widths | width of the peak (in amu units) at 20%, 50% and 80% of its total height |
| 3 | *mass accuracy* | absolute_mass_accuracy | absolute mass accuracy in amu units |
|  |  | ppm | mass accuracy in ppm |
| 4 | *peak vicinity* | subsequent_peaks_number | a number of subsequent centroids within a window of 3 peak widths (at 50% of the height) |
|  |  | subsequent_peaks_ratios | intensity ratios between the original peak and the subsequent peaks |
| 5 | *peak shape* | left_tail_auc | an integral of the difference between actual and fitted intensities on the left side of the ion peak |
|  |  | right_tail_auc | an integral of the difference between actual and fitted intensities on the right side of the ion peak |
|  |  | symmetry | a measure of peak symmetry defined as the sum of left and right tail aucs, divided by the two maximums of them |
|  |  | goodness_of_fit | reduced chi-squared, AIC and BIC of the peak fit with a Gaussian model |
| 6 | *isotopic abundance and ratios* | isotopes_ratios | intensity ratios between the main ion peak and its isotopes |
|  |  | isotopes_ratios_diffs | differences between the actual isotopes’ ratios and the expected (theoretical) ones |
|  |  | isotopes_mass_diffs | differences between the actual isotopes’ m/z values and the expected (theoretical) ones |
| 7 | *ion fragments* | fragments_ratios | intensity ratios between the main ion peak and its fragments |
|  |  | fragments_ratios_diffs | differences between the actual fragments’ ratios and the expected (theoretical) ones |
|  |  | fragments_mass_diffs | differences between the actual fragments’ m/z values and the expected (theoretical) ones |
