# Supplementary figures and images for: A system suitability testing platform for untargeted, high-resolution mass spectrometry
Source: Front Mol Biosci. 2022 Oct 11;9:1026184. doi: 10.3389/fmolb.2022.1026184 (PMC9592825; doi:10.3389/fmolb.2022.1026184)

**Figure S1.** Examples of QC features related to the expected ion peaks.

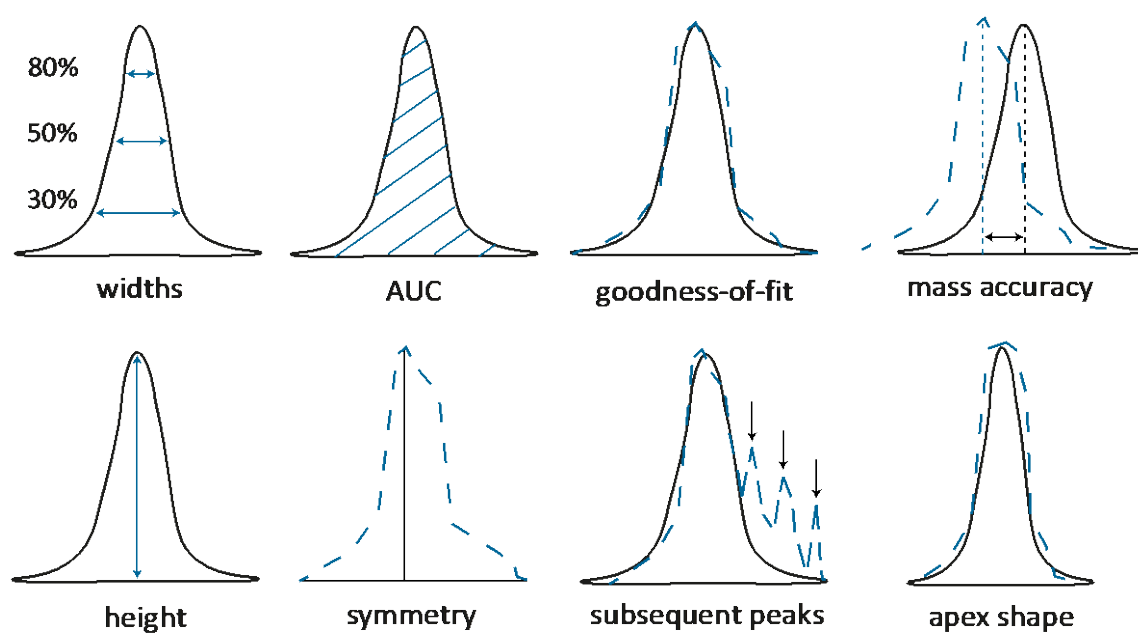

Supplement: Supplementary file 8 [file Image1.PDF]
